# Attachment of Single Teeth to a Plate, by Means of a Silicious Composition

**Published:** 1852-01

**Authors:** 


### EDITORIAL DEPARTMENT.

***Attachment of Single Teeth to a Plate, by means of a Silicious Composition.***—The readers of the Journal are all, doubtless, aware that a method, similar to that which has been occasionally practiced in France, for the last twenty-five or thirty years, for attaching single porcelain teeth to a metallic base, and the construction of artificial gums, has recently been introduced into practice in the United States. Several beautiful specimens of teeth, with artificial gums attached to plates by this means, have been sent to us by Dr. Wm. M. Hunter, of Cincinnati, Ohio, who, as is conclusively proven by documentary evidence in our possession, has been engaged in experimenting with a view to the discovery of a silicious composition for the accomplishment of this object, for some four or five years, and that his labors had been measurably crowned with success, as early as eighteen hundred and forty-nine or fifty. We have also seen a double set of teeth, with artificial gums, made by Dr. H., and attached to plates in this way, which had been worn for some time.

It also appears from evidence of the same kind, placed in our hands, that Professor J. Allen, of the same city, has been, for several years, conducting a similar series of experiments, and that they, likewise, have been crowned with the most satisfactory results. It having been stated that Professor Allen obtained the knowledge of the composition which he employs for the purpose, from a German, by the name of Steemer, affidavits have been placed in our possession, which show the allegation to be unfounded.

We have not, as yet, had an opportunity of seeing any teeth which had been mounted with Prof. A's composition, and consequently are unable to judge of the relative merits of his and Dr. Hunter's, but we have been informed that he exhibited several very beautiful specimens to the American Society of Dental Surgeons, at the meeting held in August, 1851, for which courtesy, a resolution of thanks was passed. It will also be seen by the proceedings of the Mississippi Valley Association, of Dental Surgeons, that a report recommending it to the profession, was adopted by that body, at a meeting held in Louisville, Ky., September, 1851.

With regard to the practicability of uniting American or English porcelain teeth to a metallic base, with a silicious composition, independently of any other means of attachment, and so securely as to prevent the liability of their being broken off, by mastication, we confess we have our doubts. Any composition of this sort, which will fuse at a lower temperature than that required for the fusion of the teeth themselves, must necessarily be very brittle and incapable of enduring, for any considerable length of time, we would suppose, the mechanical action to which artificial teeth are subjected. We may be mistaken, and we hope we are, but until the fact is established by practical tests, we cannot believe differently. The French porcelain teeth, less beautiful and less natural in appearance than the American and English, are capable of enduring, without injury, a much higher heat, and consequently may be united to a platina base with a composition fusible at a much higher temperature even, than that required for the fusion of the paste employed in the body of American and English mineral teeth. With such a composition, teeth may be so securely united to a *platina* base, as to preclude the liability of their being broken off by any force or action to which a dental substitute is necessarily subjected.

But, irrespective of any advantage which might be derived from a composition of this sort, as a connecting medium between the teeth and plate, we have no doubt it will prove of great practical value for the construction of artificial gums and for filling the interstices between teeth mounted in the ordinary way, and the bases upon which they are placed. This, we believe, is all that Dr. Hunter proposes to accomplish by it, though Professor Allen, from the experiments which he has made, seems sanguine in the belief that teeth can be substantially united to a plate by it.

The idea of uniting porcelain teeth to a metallic base, did not, as we have already intimated, originate in this country. The credit of that, and indeed, the discovery of a composition by which it could be done, belongs to France, the earliest nursery of modern dentistry; and, however far the United States may have outstripped other countries, within the last quarter of a century, in perfecting the practice of the dental specialty of medicine, we would not lay claim to the honor of a single discovery to which we are not justly entitled. But the composition employed in France, cannot be used in connection with teeth composed wholly, or nearly so, of spar and silex, and for the discovery of one, applicable to teeth of this kind, we believe the credit to be due to American dentists; and, so far as our information upon the subject extends, to Drs. Hunter and Allen. At any rate, the efforts of both seem to have been crowned with success. To which the priority of discovery of right belongs, we leave others to decide. It is claimed by both, and it is to be regretted that it should have given rise to the misunderstanding which seems to have grown out of it, between these gentlemen. If they cannot consent to share the honor of the discovery, and the gratitude of the profession, if it shall prove a really valuable discovery, after having been submitted to the severest practical tests, we would suggest that they lay aside all personal ill will, if they have any, towards each other, and submit their respective claims to priority in the matter, together with all the facts and evidence which may be calculated to throw light upon the subject, to friendly arbitration, by disinterested individuals, each pledging himself to abide by the decision. If the matter in dispute could be amicably adjusted in this way, it would be better for both. Private misunderstandings and quarrels are seldom productive of good, and it rarely happens that any, except the parties immediately concerned, and perhaps a few personal friends, feel much interest in such matters. After the claim to priority in the matter shall have been settled, we would then suggest that each give his discovery to the profession, charging only a reasonable fee to such as may desire personal instruction, and if it shall more than compensate for any indebtedness incurred for knowledge which he may have gained from the labors of others, the balance, we feel assured, will be more than liquidated by the gratitude of his brethren.

